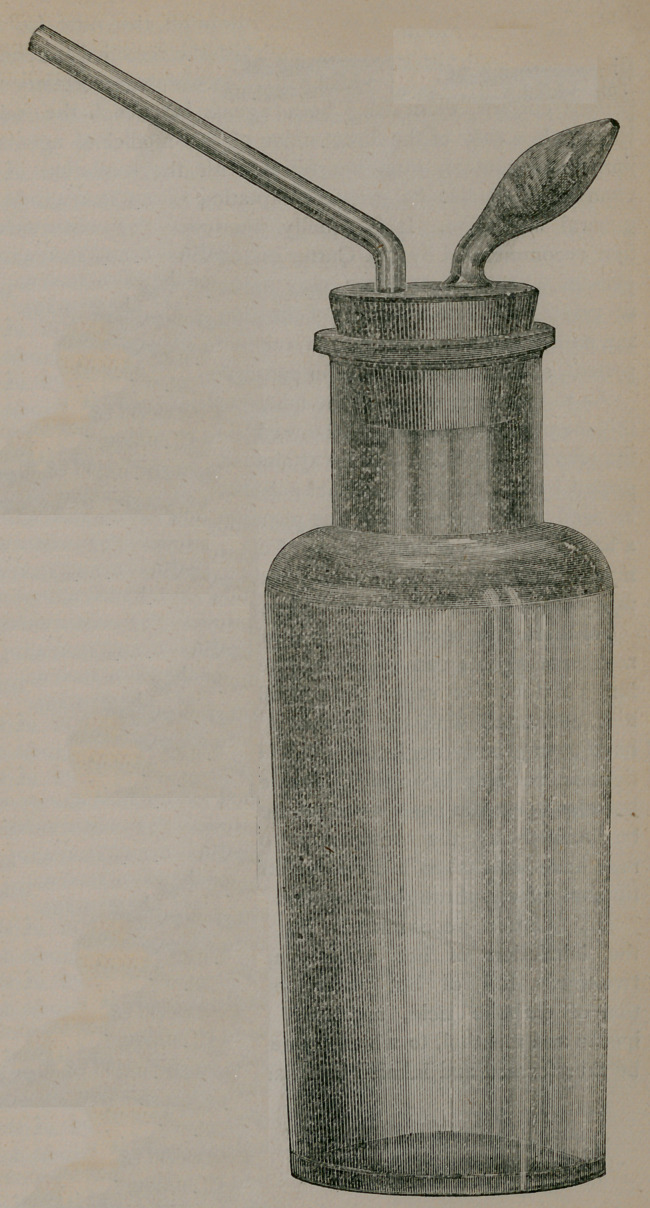# Improved Apparatus for Inhalation of Medical Agents

**Published:** 1886-02

**Authors:** Joseph. P. Logan

**Affiliations:** Atlanta, Ga.


### IMPROVED APPARATUS FOR INHALATION OF MEDICINAL AGENTS.

BY JOSEPH P. LOGAN, M. D., ATLANTA, GA.

Having had an old and suspended interest in the application of medicinal agents by inhalation revived by an article of Dr. Ephraim Cutter, of New York, in the August number of the *Physician's Magazine*, describing an apparatus for the ready formation and inhalation of the nascent chloride of ammonium, accompanied with a cut of the same, I have been induced to take up a course of experiment pursued, to a slight extent, some years since.

While I have a number of patients, under this plan of treatment, affected variously with chronic nasal, post-nasal, pharyngeal, laryngeal and bronchial catarrh, I am not prepared at present to detail cases or to speak otherwise than in generally favorable terms as to what is likely to be obtained by this mode of medication in the obstinate affections alluded to, the almost uniformly unsatisfactory results of treatment in which, I believe to be generally admitted.

What I propose at present is simply to call attention to what I suppose to be an improvement in the apparatus for the generation and application of the nascent muriate of ammonia by inhalation, recommended years ago by Dr. Lewis Elsberg, of New York, in chronic affections in the air passages, and recently by Dr. Ephraim Cutter, of New York, in acute affections of a similar character, and in addition, to suggest some other agents which may profitably and possibly, to a greater extent, be found beneficial in this treatment of disease.

The accompanying cut will show, as in the case of Dr. Cutter's inhaler, an eight-ounce reservoir of glass, larger or smaller, as may be preferred. A stopper of rubber (instead of wood as in his case) with two perforations, one for the inhaling tube and the other for a bulbous tube, which is packed with strips of sponge, which is used for the ammonia instead of the hook of iron upon which the sponge is impaled, to be placed in the reservoir, as in the case of Dr. Cutter's inhaler. The hydrochloric or muriatic acid of full strength, to the extent of one or two teaspoonsful, is put into the reservoir and the sponge in the bulb receives from beneath the stopper ten or fifteen drops of strong liquor ammonia most readily by a minim dropper. By blowing gently through the inhaling tube, as in Cutter's instrument, into the reservoir, a current is formed and the vapors mix and a cloud of crystals is formed which escapes through the bulbous tube. The nascent salt thus formed is then ready for inhalation through the tube for that purpose. The supposed advantages of the modified instrument are, greater simplicity of construction, absolute certainty of a perfect current, embracing both agents by which the salt is formed, less risk of the direct union of the medicinal agents or immediate contact, thus interfering with the evolution of the vapory salt, as also its greater adaptation as an instrument for general inhalation. It is equally adapted to the passive inhalation recommended by Dr. Cutter in the case of children or feeble persons where an attendant may fill an apartment with a cloud of cyrstals or blow a current of the same directly into the face of the patient, or still better into a tube held in the mouth of the patient, such as is found accompanying an atomizing apparatus.

As to the continuance of the inhalation at each time it is used, this must be determined by the medical adviser, depending upon the effect and the object to be attained, but as this salt, as suggested by Dr. Cutter, is neither poisonous nor deleterious, it may be used at the discretion of the physician from a few minutes to a half hour several times daily. For the present, I usually direct my patients to use it for fifteen minutes three times a day, but in many cases it could no doubt be greatly prolonged to advantage.

As there now seems to be no doubt that this and many other medicinal agents can penetrate the air passages, and even the lungs in a state of vapor, the possibilities, and even the probabilities, connected with the inhalation of remedies would seem to have been greatly neglected and to offer a field worthy of investigation, not only as to their local effects in modifying nutrition, but also in certain cases as to the beneficial effects which might be obtained by the destruction of injurious agents which may have been taken in from the atmosphere or by their constitutional effects, through absorption into the system.

Dr. Cutter recommends his apparatus as valuable for the inhalation of hot water vapor by filling the reservoir two-thirds full of hot water, or medicating by the addition of carbolic acid, cresolene, poppy leaves, hops, mullein, ipecac and menthol, to which I would add other salts, which may be thus formed and inhaled in a nascent state, which may prove even more beneficial than the muriate of ammonia, viz.: Bromide of ammonium, iodide of ammonia, carbolate of ammonia, carbolate of ammonia and iodine, carbolate of iodine and benzoate of ammonia. It may also be beneficially used for the inhalation of nitrate of amyl, chloroform, ether, bromic ether, camphor, turpentine, essential oils, iodic ether, iodic, benzoic, salicylic and hydrocyanic acids and oxygen. In the case of some of these the addition of heat may be required, either by placing the reservoir in hot water or by the use of a spirit lamp.

In conclusion, I desire to acknowledge my indebtedness for the instrument to which I have called attention to the ingenuity of Mr. A. Sidney Rauschenberg, a promising young chemist and pharmacist of this city, in connection with the pharmaceutical establishment of Th. Schumann, where the inhaler may be obtained, with full and special directions as to the manner of using it in the various ways suggested, it being necessary to observe some special chemical precautions in the generation and application of some of the agents mentioned.

## Figures and Tables

**Figure f1:**